# MPT64 antigen-induced immune responses as a novel diagnostic tool for tuberculosis

**DOI:** 10.1128/spectrum.03381-24

**Published:** 2026-03-25

**Authors:** Jian Hu, Huan Wu, Fan Su, Xia Ren, Chenxu Wang, Wendan Mei, Yicheng Fang, Xiaolei Tang, Yufeng Wen

**Affiliations:** 1 School of Public Health, Wannan Medical College 74649, Wuhu, Anhui, China; 2 School of Laboratory Medicine, Wannan Medical College 74649, Wuhu, Anhui, China; 3 The Second Affiliated Hospital of Wannan Medical College Translational Medical Center 586378, Wuhu, Anhui, People’s Republic of China; City of Hope Department of Pathology, Duarte, California, USA

**Keywords:** tuberculosis diagnosis, MPT64 antigen, CLE fusion protein

## Abstract

This study aimed to evaluate novel antigen targets from the region of difference (RD) in Bacillus Calmette–Guérin (BCG) for improving tuberculosis (TB) diagnosis. Seven RD region antigens of *Mycobacterium tuberculosis* (*M.tb*) were expressed in *Escherichia coli,* and their immunogenicity was assessed by enzyme-linked immunosorbent assay (ELISA) in immunized mice. MPT64 emerged as the most potent secretory protein, inducing the highest interferon-gamma (IFN-γ) production in mouse spleen cells. Both MPT64 alone and in combination with CLE (a fusion protein of early secretory antigenic target-6 [ESAT-6] and culture filtrate protein-10 [CFP-10]) significantly enhanced IFN-γ production in peripheral blood mononuclear cells (PBMCs) from TB patients, demonstrating high diagnostic sensitivity and specificity. TB patients exhibited markedly elevated MPT64-specific antibodies, with the immunoglobulin G4 (IgG4) subclass showing optimal detection performance. Skin tests successfully differentiated mice immunized with H37Rv from those immunized with BCG and phosphate-buffered saline (PBS), while immunized rabbits produced high levels of MPT64 antibodies. The peptide TDPLPVVFPIVQGE from MPT64 elicited the strongest IFN-γ production and skin reaction, while TQAVVLKVYQN showed the strongest antibody binding. These results indicate that MPT64 is a specific M. tuberculosis antigen with significant potential for TB diagnosis through multiple approaches, including IFN-γ release assays, skin tests, and serological detection, particularly when combined with CLE for enhanced diagnostic performance.

**IMPORTANCE:**

MPT64 is a protein from the tuberculosis (TB) bacterium that triggered the strongest immune response among seven new antigens tested. It shows great promise for making TB diagnosis more accurate. MPT64 worked well in different test formats, including blood tests that measure a key immune molecule (interferon-gamma), tests that detect TB antibodies, and skin tests like the TB skin test. It also performed strongly when combined with another TB antigen fusion called CLE (a merger of two other TB proteins, early secretory antigenic target-6 [ESAT-6] and culture filtrate protein-10 [CFP-10]). These results suggest that adding MPT64 to current TB tests could help doctors detect TB infection more easily and reliably.

## INTRODUCTION

Tuberculosis (TB) is an infectious disease caused by *Mycobacterium tuberculosis* (*M.tb*). According to the Global Tuberculosis Report 2023, there were an estimated 10.6 million new cases worldwide in 2022 (1), with China being one of the high-burden countries for TB, accounting for 7.1% of the global total. The global incidence rate of TB is 133 per 100,000, which has increased by 3.9% compared to the period of 2020–2022 (2). The challenges of control have been exacerbated by the emergence of multidrug-resistant tuberculosis, extensively drug-resistant strains, and co-infections with HIV (3). Therefore, the diagnosis of latent tuberculosis infection (4) is particularly important.

Current diagnostic approaches for TB include the tuberculin skin test (TST), sputum smear acid-fast staining, radiological X-ray imaging, and sputum culture (5). While these methods have historically met basic diagnostic needs, their limitations have become increasingly apparent amid the prevalence of diseases, such as HIV and COVID-19, as well as the rise in drug-resistant TB cases. Emerging diagnostic technologies, such as automated digital microscopy algorithms and novel molecular diagnostic techniques (6), offer new perspectives but remain immature and costly. Serological testing and IFN-γ release assays (IGRAs) (7) serve as important auxiliary tools. IGRAs diagnose *M.tb* infection by measuring the level of interferon-gamma (IFN-γ) produced by peripheral blood lymphocytes. The T-SPOT.TB assay, an IGRA that utilizes early secretory antigenic target-6 (ESAT-6) and culture filtrate protein-10 (CFP-10) peptides as stimulating antigens, has shown variable sensitivity and specificity for detecting active TB (8, 9). Advancements in molecular biology have facilitated the discovery of new antigenic targets, particularly within the region of difference (RD) identified through gene chip technology (10). The 16 regions within the RD contribute to the specificity of TB diagnosis. Notably, the MPT64 protein from the RD2 region has demonstrated the ability to differentiate between TB patients and non-patients in preliminary tests and is anticipated to enhance combined diagnostic methods (11).

To improve TB diagnostic efficacy, we aim to express selected genes from the RD region and identify highly expressed and stable RD region antigenic proteins. Additionally, we will incorporate the CLE antigen protein, previously utilized in detection kits, as a standard control to develop methodologies and evaluate the performance of RD region antigenic protein detection. Concurrently, we seek to identify dominant epitopes of RD region proteins to inform the preparation of peptides for commercial detection kits, thereby supporting the modern detection and control of TB.

## MATERIALS AND METHODS

### Participants

A total of 40 serum and peripheral blood mononuclear cell (PBMC) samples were collected from patients with active pulmonary TB at the First Affiliated Hospital of Wannan Medical College, China, between May 2022 and January 2024. Only culture-confirmed samples were included in the TB group. Additionally, 50 serum and PBMC samples from healthy donors (HDs), including Bacillus Calmette–Guérin (BCG)-vaccinated individuals, were obtained from the hospital’s medical examination center. The inclusion criteria for the tuberculosis group were (i) the diagnostic criteria for pulmonary tuberculosis in the Health Industry Standards of the People’s Republic of China—Diagnostic Criteria for Pulmonary Tuberculosis (WS288-2017); (ii) no history of other lung diseases; and (iii) primary tuberculosis patients who have been treated for the first time. Inclusion criteria for the healthy control group: strictly paired with the case group according to age, gender, and ethnicity. Exclusion criteria for the tuberculosis group and healthy control group: other types of tuberculosis infection, such as intestinal tuberculosis, spinal tuberculosis, etc., history of tumor cancer, suffering from autoimmune diseases, such as systemic lupus erythematosus, rheumatoid arthritis, autoimmune liver disease, etc., and inflammatory diseases caused by bacterial infections other than *M.tb*. The study was approved by the hospital’s ethics review board, and all subjects provided informed consent (The Medical Ethics Committee of Wannan Medical College No. 2024163).

### Enzyme-linked immunospot assay

In the mouse enzyme-linked immunospot (ELISpot) assay, female BALB/c mice (6–8 weeks old) were immunized via tail vein injection with inactivated *M.tb* (iH37Rv, heated at 65°C for 1 hour) at a dose of 100 µL of 10⁹ iH37Rv/mL daily for seven consecutive days. Fifteen days after the final injection, the mice were sacrificed, and splenocytes were isolated. Adjust the cell volume to 1 × 10^6^ CFU/mL. The splenocytes were stimulated with 5 µg/mL of each RD recombinant protein in EZ-Culture serum-free medium (DAKEWE Biotechnology, Beijing, China) for 24 hours before being added to IFN-γ monoclonal antibody-coated plates.

In the human ELISpot assay, PBMCs (2.5 × 10⁵ cells per well) from TB patients and healthy donors were stimulated with MPT64, other RD proteins, CLE protein (5 µg/mL), or CFP10-ESAT6 peptides (20 µL, a 1:1 mixture provided in the clinical T-SPOT.TB kit) for 24 hours. IFN-γ levels were measured using the human IFN-γ T-SPOT.TB kit (DAKEWE Biotechnology) and quantified as spot-forming units (SFUs) using an immunospot image analyzer (BioReader-E, BioSYS, Germany).

Positive controls included phytohemagglutinin (PHA), CFP10, ESAT6, and CLE fusion proteins, while phosphate-buffered saline (PBS) served as the negative control. All samples were tested in duplicate wells across three independent experiments. Results were classified as positive (>6 SFUs), negative (<6 SFUs), or indeterminate (if >10 SFUs were present in PBS control wells and/or <20 SFUs were present in PHA-positive control wells).

### Measurement of delayed-type hypersensitivity response

Female BALB/c mice (6–8 weeks old) were immunized either by intravenous injection of 10⁸ CFU of heat-inactivated *M.tb. H37Rv* seven times or by intradermal injection of 2 × 10⁸ CFU of BCG. Twenty-eight days post-immunization, skin tests were performed by intradermally injecting 10 tuberculin units (TU) of purified protein derivative (PPD) or 1 mg of recombinant MPT64 protein into the hair-removed flank region of the host. The diameter of induration was measured 24 hours later, with diameters exceeding 5 mm considered positive.

### Determination of T-cell and B-cell epitopes

T-cell epitopes: thirteen peptides encompassing the entire primary structure of MPT64 were synthesized. To evaluate T cell dominant epitopes, an IFN-γ enzyme-linked immunosorbent assay (ELISA) was conducted following the manufacturer’s protocol (DAKEWE Biotechnology, Beijing, China). Mouse splenocytes from *M.tb. iH37Rv*-immunized female BALB/c mice and PBMCs from TB patients (ages 19–65) were seeded at 2.5 × 10⁵ cells per well and stimulated with 5 µg/mL of each MPT64 peptide for 24 hours. IFN-γ release was quantified using a standard cytokine curve. All samples were tested in duplicate across three independent experiments. To confirm the identified T cell epitopes, sensitized female BALB/c mice received intradermal injections of 1 mg each synthetic peptide, and subsequent skin reactions were assessed as previously described.

B-cell epitopes: B cell dominant epitopes were identified using ELISA. Polystyrene microtiter plates were coated with 1 µL of each MPT64 synthetic peptide (5 µg/mL) and incubated with anti-MPT64 polyclonal antibodies (1:10,000 dilution), followed by HRP-conjugated goat anti-rabbit IgG (1:4,000 dilution). Color development was achieved by adding 100 µL of TMB substrate (0.2 mg/mL, Sigma-Aldrich) and terminating the reaction with 50 µL of 2 M H₂SO₄. Optical density (OD) was measured at 450 nm using a PerkinElmer 2030 ELISA reader. Additionally, B cell epitopes were validated by dot blot analysis. Circular depressions were created on PVDF membranes by pressing with the inverted blunt end of a 1 mL pipette tip. These served as wells to confine protein solutions within defined boundaries during 37°C drying in a controlled environment, preventing uncontrolled spreading and ensuring consistent solidification within the circular zones. Each MPT64 synthetic peptide (20 µg) was spotted onto methanol-activated PVDF membranes, blocked with 5% skim milk, and incubated with anti-MPT64 polyclonal antibodies (1:10,000 dilution), followed by HRP-conjugated goat anti-rabbit IgG (1:4,000 dilution). Color development was analyzed using the Dot Blot Analyzer toolset for Image J.

### Serum antibody measurement by ELISA

Ninety-six-well microtiter plates were coated with individual RD proteins and CLE fusion protein (10 µg/mL, 100 µL per well) in coating buffer and incubated at 4°C overnight. After four washes with PBS containing 0.05% Tween 20 (PBST), the plates were blocked with 200 µL/well blocking buffer (2% bovine serum albumin [BSA] in PBST) at 37°C for 1 hour. Following washing, 200-fold diluted human serum samples were added and incubated at 37°C for 1 hour. The plates were thoroughly washed and then incubated with HRP-conjugated goat anti-human IgG, IgM, IgG2, and IgG4 antibodies (1:4,000 dilution) at 37°C for 30 minutes. Subsequently, 100 µL/well of TMB substrate was added. The reaction was stopped by adding 50 µL of 2 M H₂SO₄, and optical densities were measured at 450 nm within 10 minutes using a PerkinElmer 2030 multilabel ELISA reader. All samples were tested in duplicate wells across three independent experiments.

### Statistical analysis

Data were analyzed using SPSS software. Differences between groups were evaluated using one-way ANOVA and independent-sample *t*-tests, with *P* < 0.05 considered statistically significant. Receiver operating characteristic (ROC) curves were generated using GraphPad Prism software, and the area under the curve (AUC) was calculated to assess diagnostic performance.

## RESULTS

### Expression and identification of some genes in the RD

**Fig 1:**
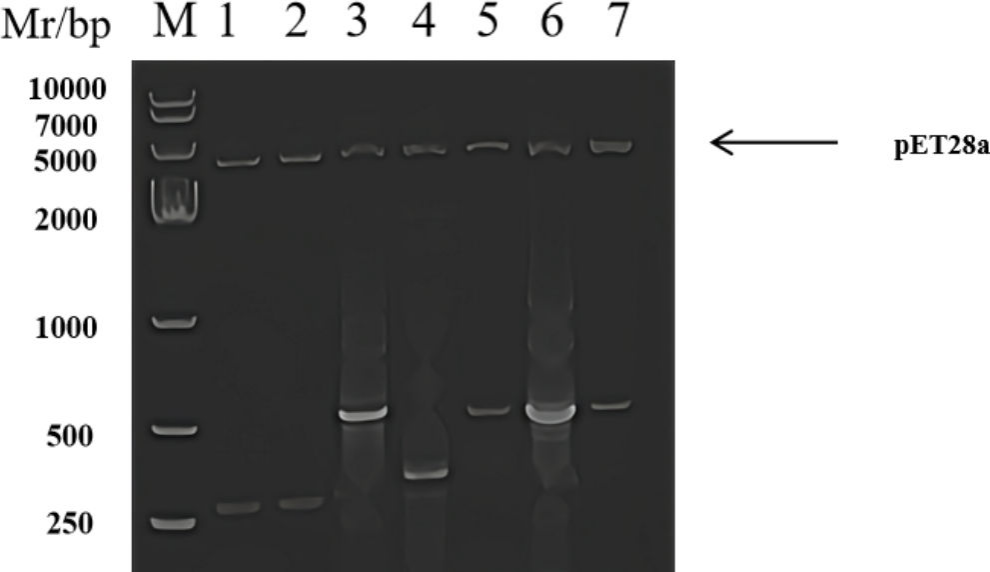
Double digestion identification of the constructed recombinant plasmids (1–7 represent RD genes Rv3874, Rv3875, Rv1773, MPT 63, CLE, MPT 83, and MPT64. Size: 260, 270, 500, 390, 500, 470, and 615 bp, respectively; M represents Marker).

Gene sequences of highly expressed RD region genes were retrieved from NCBI. Primers were designed using specialized software and synthesized by Shanghai Biosynthesis for PCR amplification. Correct PCR products were confirmed via agarose gel electrophoresis, then ligated into a prokaryotic expression vector. Successful ligation was verified through double enzyme digestion, ensuring all genes were accurately inserted. The concentration of each RD protein is 10 ug/mL (Fig. 1).

### Expression and identification of proteins in the RD

**Fig 2:**
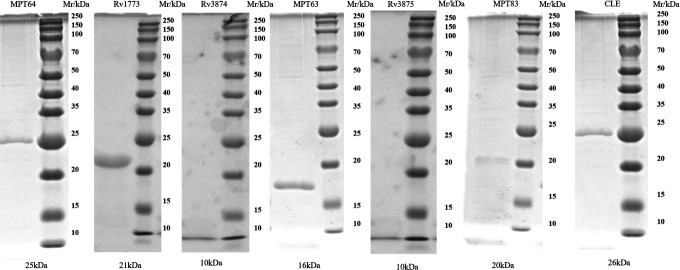
Identification of SDS-PAGE of seven proteins, including MPT64.

Successfully ligated RDs-pET28a recombinant plasmids were introduced into competent *Escherichia coli* DH5α for amplification, and then introduced into competent *E. coli* BL21 for expression protein. After IPTG induction, the protein was purified by washing and eluted through a Ni-NTA Agarose column, and the target protein was identified by SDS-PAGE. The results showed protein bands at the corresponding molecular mass after induced expression of the recombinant protein and were correctly identified by purification (Fig. 2).

### MPT64 protein secretability identification and IFN-γ detection of stimulated lymphocyte production

**Fig 3:**
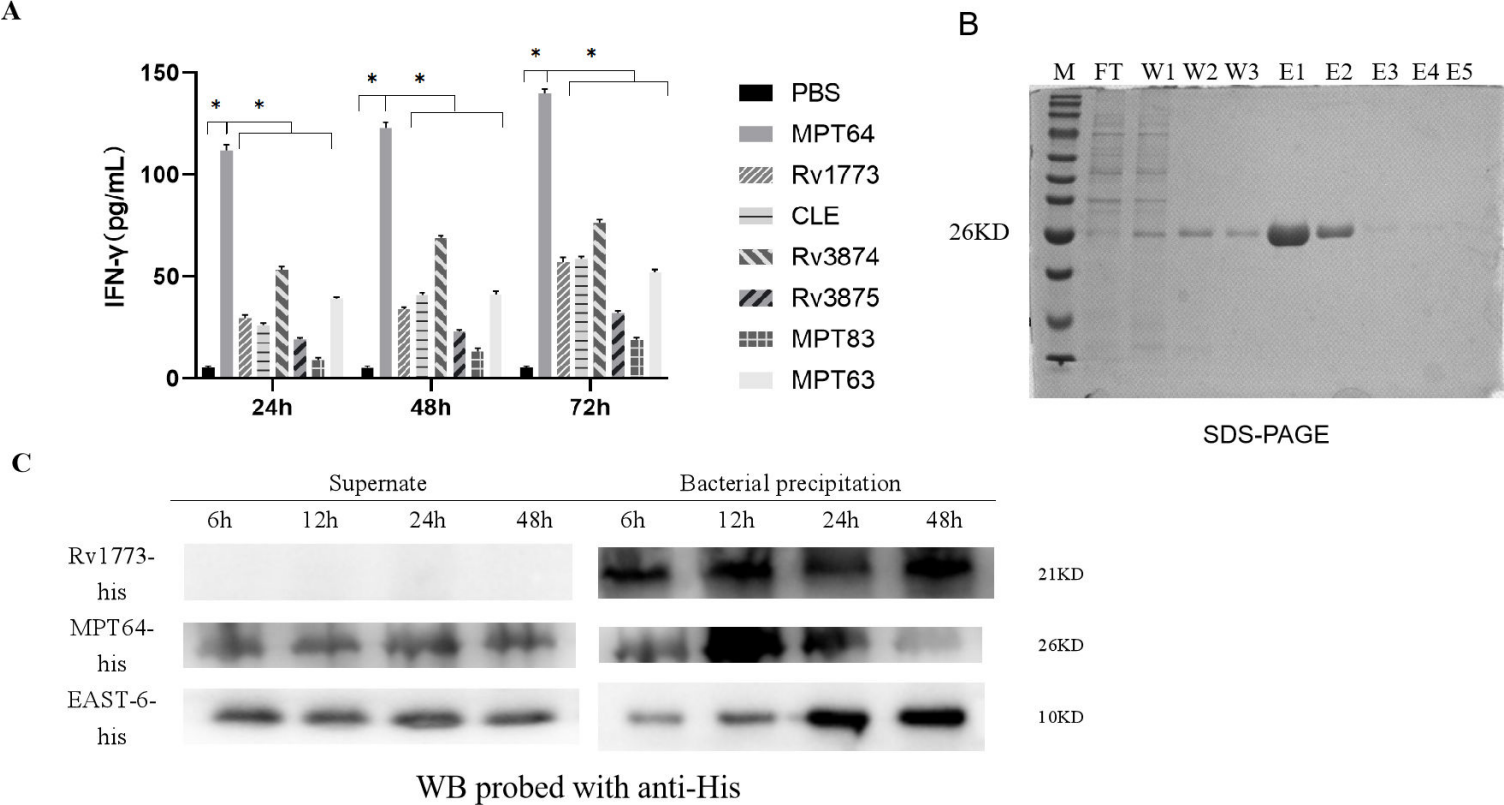
IFN-γ levels produced by lymphocytes stimulated by secreted-type proteins. (**A**) ELISA analysis of IFN-γ levels in splenocytes of immunized mice at different times (*P < 0.001 compared with MPT64 group). (**B**) Identification by the SDS-PAGE of MPT64. (**C**) WB identification of supernatant versus bacterial precipitate at different time points after MPT64 induction expression.

To screen the RD proteins with high expression of IFN-γ, the level of IFN-γ produced by mouse splenocytes stimulated by each antigen protein was detected by ELISA. The results showed that MPT64 protein stimulated IFN-γ expression in mouse spleen cells and stimulated the strongest level of IFN-γ production among seven RD proteins (*P* < 0.001) (Fig. 3).

### The MPT64 protein has a diagnostic utility for cellular immunity

**Fig 4:**
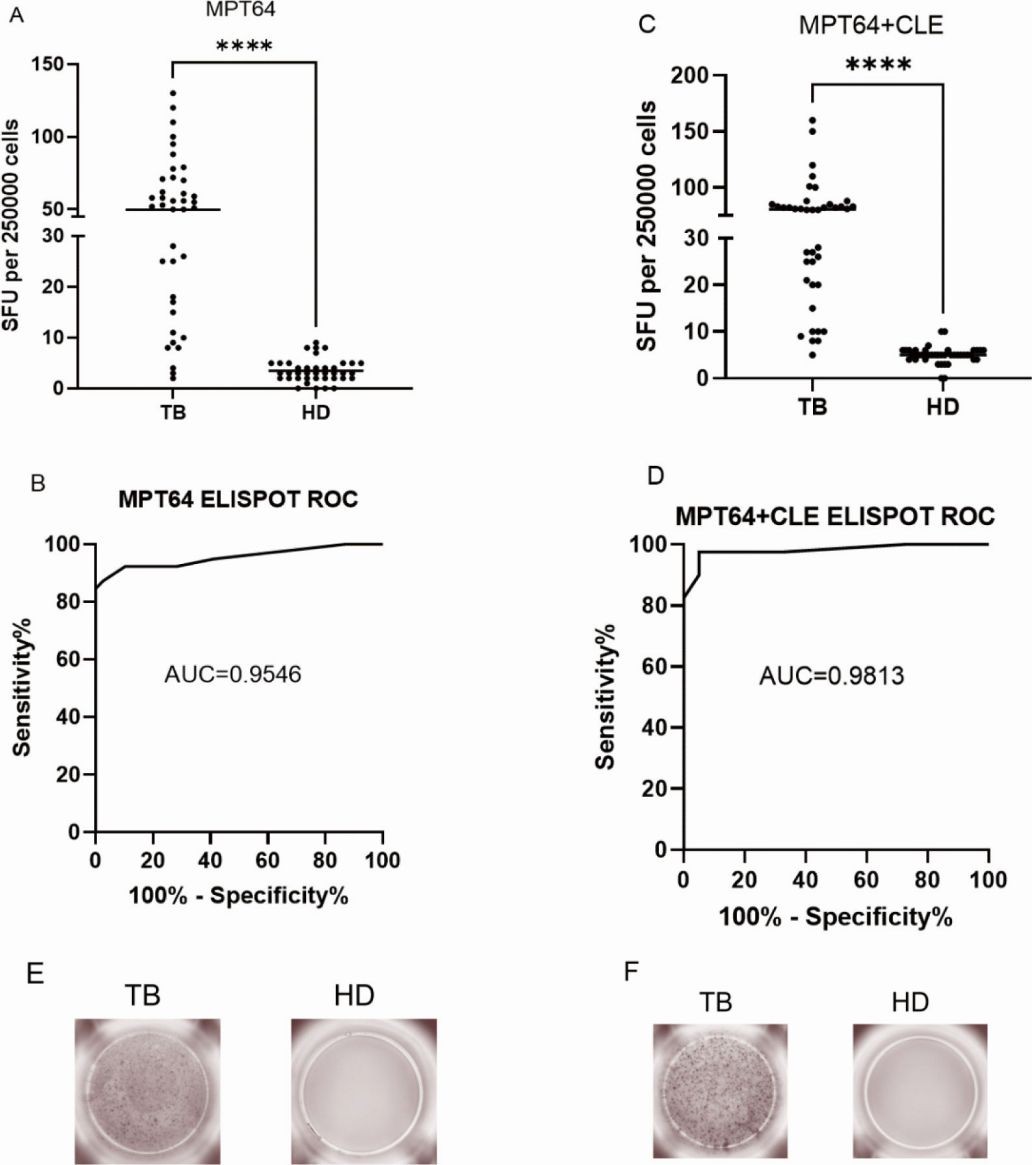
The release of IFN-γ from both MPT64 and MPT64+CLE was much higher in active pulmonary TB patients than in the healthy control population. (**A**) IFN-γ levels of active tuberculosis and healthy control MPT64. (**B**) The ROC curve of the ELISpot for MPT64. (**C**) IFN-γ levels of active tuberculosis and healthy control MPT64+CLE. (**D**) The ROC curve of the ELISpot for MPT64+CLE. (**E**) Representative plot of MPT64 protein as antigen-stimulated PBMC in TB humans and healthy controls. (**F**) Representative plot of MPT64+CLE protein as antigen-stimulated PBMC in TB humans and healthy controls. “****” indicates a statistically significant difference.

The ELISpot assay revealed that stimulating PBMCs with MPT64 alone or combined with CLE significantly increased IFN-γ-producing cells in TB patients compared to healthy donors. ROC analysis showed that MPT64 achieved an AUC of 0.9546 for TB diagnosis, and combining it with CLE further improved the AUC to 0.9813, enhancing both sensitivity and specificity (Fig. 4).

The MPT64 protein stimulated PBMCs from active TB patients and healthy donors with a sensitivity of 87.5%, specificity of 94.0%, and Youden’s index (YI) of 0.815. When combined with the CLE antigen, sensitivity and specificity increased to 95.0% and 98.0%, respectively, with a YI of 0.930, effectively distinguishing TB patients from non-patients (Table 1).

**TABLE 1:** ELISpot detection in TB patients using MPT64 and MPT64+CLE^*a*^

	ELISpot (MPT64)		Total	ELISpot (MPT64+CLE)		Total
	+	−		+	−	
TB	35	5	40	38	2	40
HD	3	47	50	1	49	50
Total	38	52	90	39	51	90

^*a*^ The cut-off of MPT64/MPT64+CLE ELISpot was 6 SFUs/25,000 PBMC cells.

Compared to the T-SPOT.TB assay (ESAT6-CFP10 peptides), the MPT64 ELISpot demonstrated a kappa value of 0.805 (*P* < 0.001), indicating significant agreement. This suggests that MPT64 ELISpot can serve as a reliable clinical alternative to the T-SPOT.TB assay (Table 2).

**TABLE 2:** MPT64 ELISpot vs clinical T-SPOT TB detection

Test methods		ELISpot (MPT64)		Total
		+	−	
T-SPOT.TB	+	33	0	33
	−	2	5	7
Total		35	5	40

### Serum antibody detection for TB diagnosis

**Fig 5:**
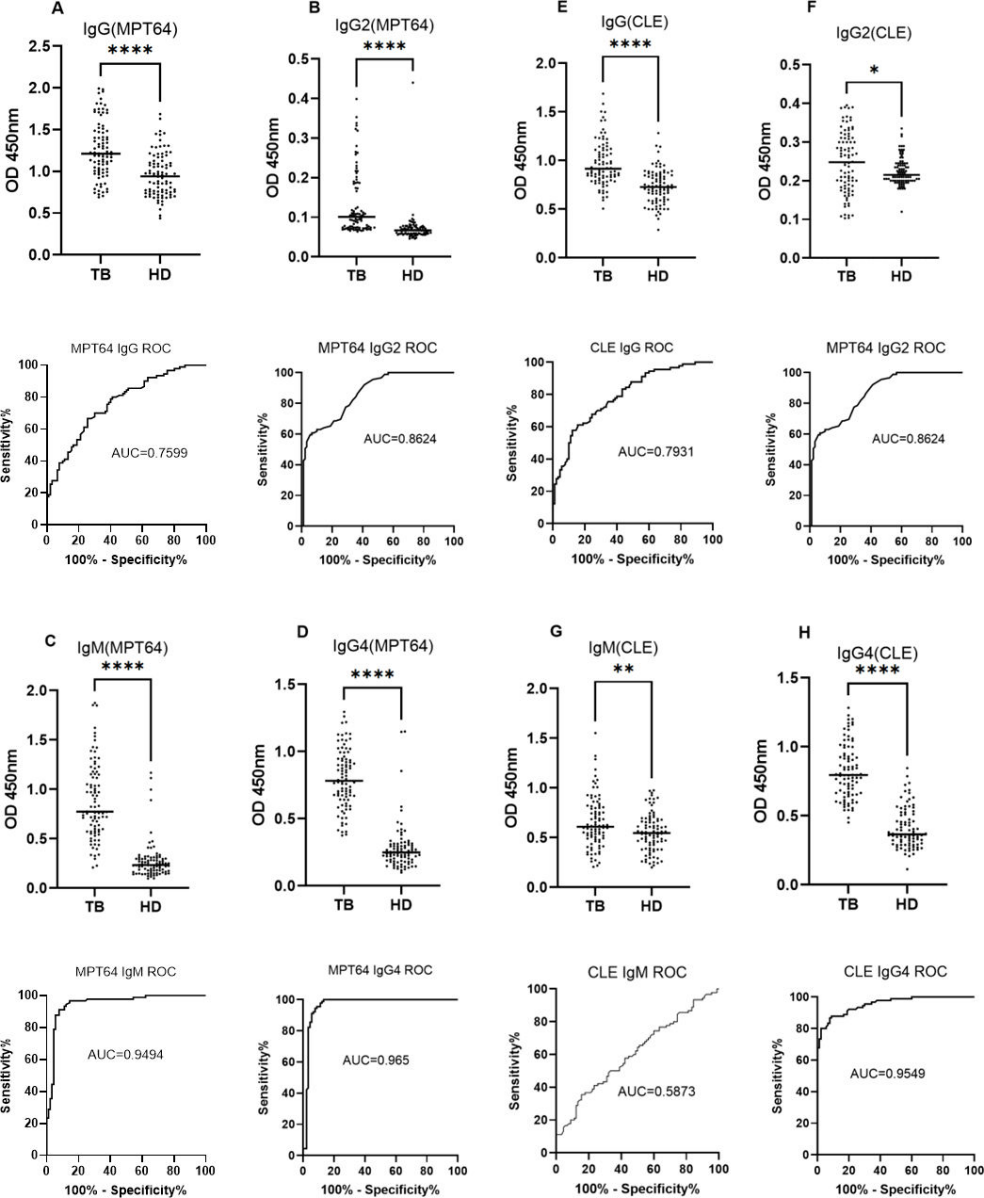
MPT64 and CLE-specific antibody levels in TB human versus healthy populations (**A–H**). "*", "**", and "****" indicates a statistically significant difference.

In this study, we also found that MPT64-specific antibodies, IgG, IgG2, IgM, and IgG4 in human tuberculosis serum were significantly higher than those in healthy people, and the detection of serum antibodies specific for CLE protein was used as a positive control. In this study, it was found that IgG4 tested the best effect, and each statistical indicator was significantly better than its antibody subtype and each index of the CLE group (Fig. 5).

### The optimal working concentration of MPT64 antigen serum dilution 1:800 was 10 ng/mL

**Fig 6:**
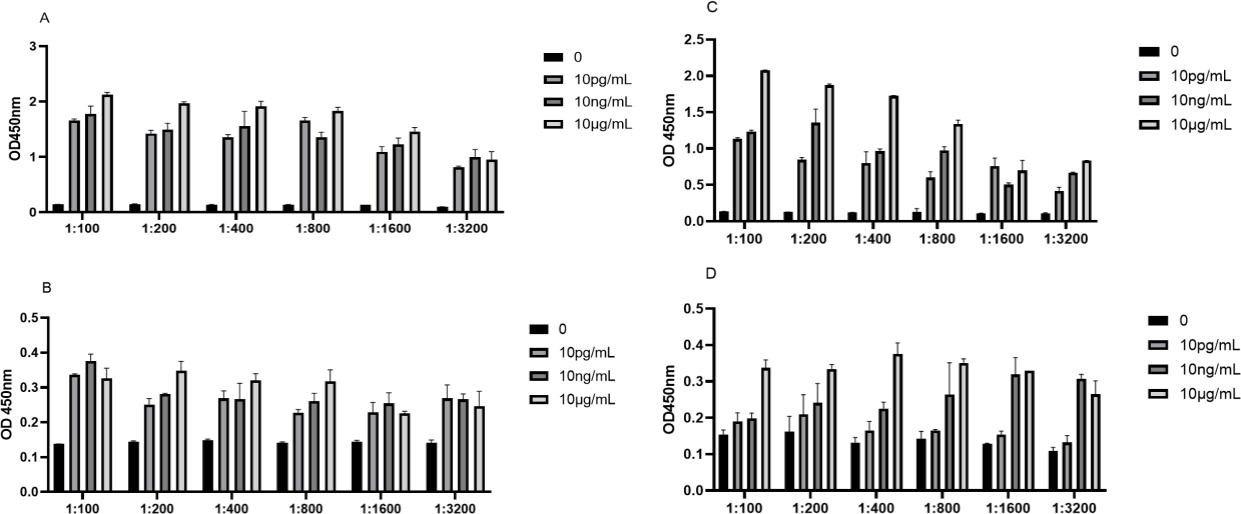
The checkerboard ELISA determines the optimal working concentration of the antigen. (**A**) OD value of TB serum and ELISA of MPT64 antigen-coated test. (**B**) OD of HD serum and ELISA of MPT64 antigen. (**C**) OD of ELISA with CLE antigen coated. (**D**) OD value of the ELISA of HD serum and CLE antigen coating.

In 0 pg/mL, 10 pg/mL, 10 ng/mL, and 10 µg/mL antigen protein-coated ELISA plates, 1:100, 1:200, 1:400, 1:800, 1:1,600, and 1:3,200 fold dilutions, with CLE protein as a control, OD_TB_/OD_HD_. The maximum value to select the optimal working concentration of the antigen. The experimental results show that the lowest working concentration of antigen can reach the level of 10 pg/mL. Antigen MPT64 and “star antigen” CLE are at the ng level. The OD_TB_/OD_HD_ can obtain the maximum value. The OD was achieved for MPT64 at a serum dilution of 1:800 with an antigen concentration of 10 ng/mL OD_TB_/OD_HD_. The maximum value, 6.53, is the optimal working concentration of the antigen. CLE achieved an OD at a serum dilution of 1:100 with an antigen concentration of 10 ng/mL. The maximum OD_TB_/OD_HD_ value of 6.20, for the optimal working concentration of the antigen (Fig. 6).

### MPT64 induced late-onset hypersensitivity reactions (DTH) in mice

**Fig 7:**
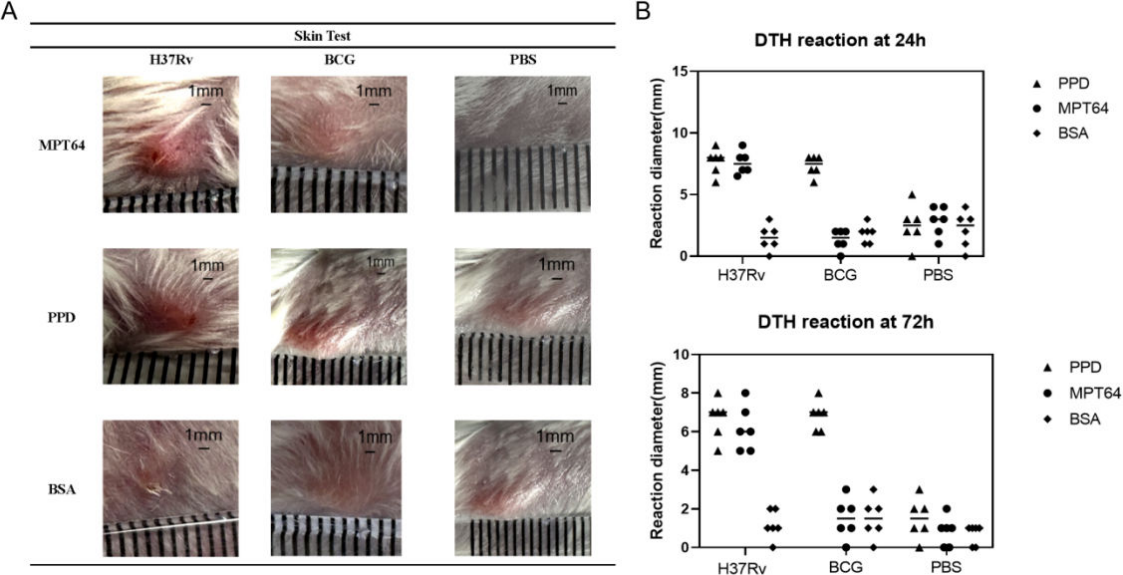
MPT64 can induce late-onset hypersensitivity in mice. Eighteen Balb/c female mice were divided into three groups of six and immunized seven times with inactivated H37Rv, BCG, and PBS. Then, the PPD, MPT64, and BSA were used as antigens and observed for 24 to 72 hours. Erythroid nodules >5 mm were positive for delayed-type hypersensitivity (DTH). (**A**) Solid representation of typical erythematous nodules 24 hours after antigen injection in mice of each group. (**B**) Size of DTH erythema nodules after 24 hours (top) and 72 hours (down) of H37Rv, BCG, and PBS injection. The column ruler has a minimum of 1 mm scale.

The MPT64 protein, as an antigen, can cause delayed hypersensitivity, similar to the commonly used tuberculin PPD. Differently, PPD is not able to distinguish between early H37Rv and infection with BCG. However, MPT64, as an antigen, was able to distinguish H37Rv infection from BCG inoculation (Fig. 7).

### Preparation and titer detection of MPT64 and rabbit polyclonal antibody in CLE

**Fig 8:**
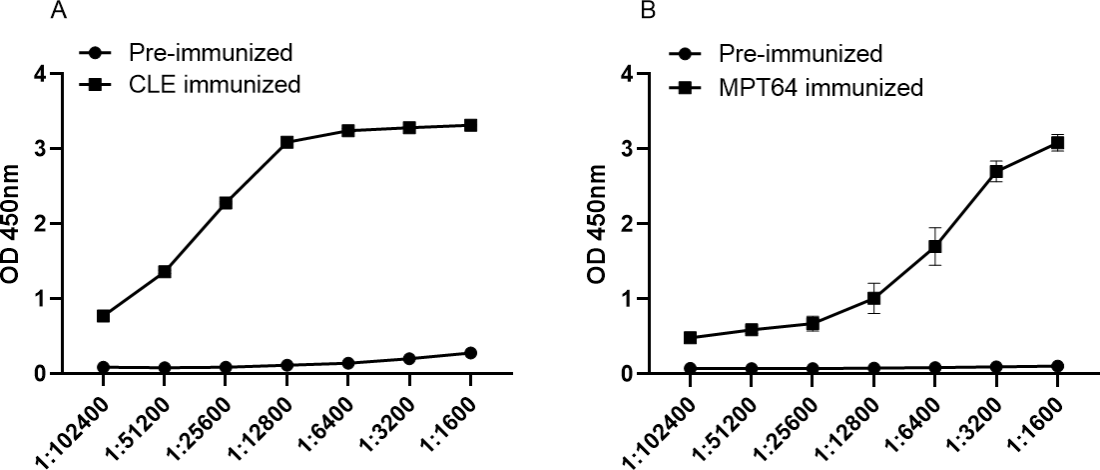
Preparation and potency testing of rabbit polyclonal antibodies against CLE (**A**) and MPT64 (**B**).

The SPF grade New Zealand rabbits were selected, with 1 mL of blood drawn from the ear margin vein, and the serum was centrifuged as a control before antigen immunization. Antigen was immunized three times with 1 mg of antigen protein with Freund’s once a week. After immunization, the antibody titer was determined by ELISA. After the titer reached 1:102,400, rabbits were put into the carotid artery. CLE antibody OD_450nm_ dilution 1:102,400/preimmunization OD_450nm_ was 9.83, and MPT64 antibody. 1:102,400 dilution OD_450nm_ preimmunization OD_450nm_ was 2.40 greater than the bound value of 2.1. The quality of the antibody can meet the needs of the experiment (Fig. 8).

### T cells of MPT64 antigen, B cell predominant response epitope MPT64 (152-165), and MPT64 (109-119)

**Fig 9:**
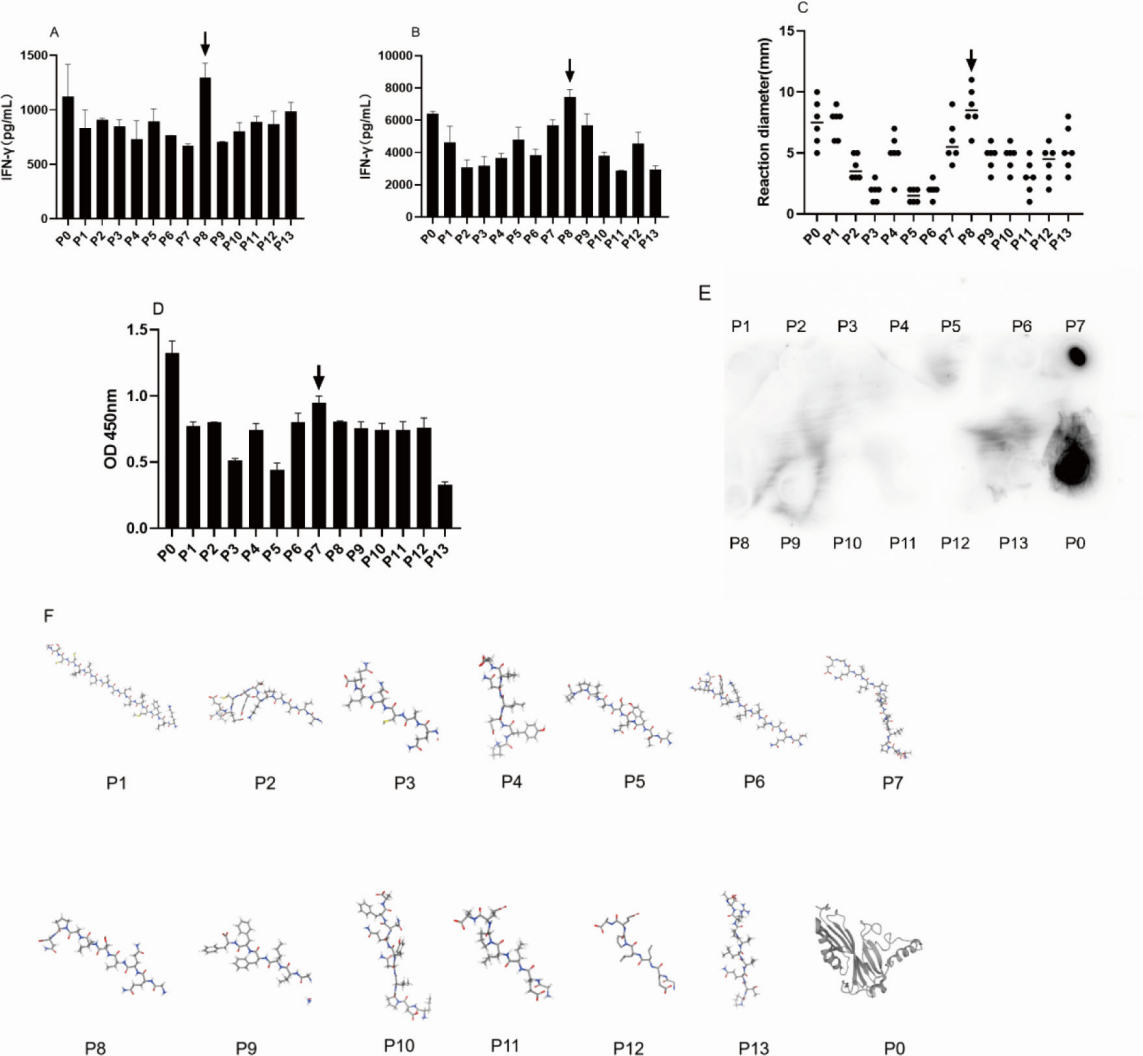
Dominant epitopes in the T and B cells of the MPT64 protein. (**A**) ELISA measurement of IFN-γ release of each peptide and the complete sequence of MPT64. (**B**) The ELISA measurement of IFN-γ release from TB human PBMC stimulation by each peptide and the MPT64 complete sequence. (**C**) DTH of each polypeptide and the complete sequence of MPT64 in the mouse. (**D**) ELISA absorbance of the affinity of each polypeptide and the complete sequence of MPT64 to the MPT64 rabbit polyclonal antibody. (**E**) Spot hybridization shows the affinity of each polypeptide and the complete MPT64 sequence with the MPT64 rabbit polyclonal antibody. (**F**) Schematic diagram of the 3D predicted structure of each polypeptide, as well as the complete sequence of MPT64.

To better grasp the immunogenicity of the MPT64 protein. According to the sequence of the MPT64 gene provided by NCBI, it was translated into protein through the protein translation function of DNAMAN software, and then the MPT64 antigen protein was divided into 13 peptides according to the predicted epitope function, hydrophobicity, and hydrophilicity (Table 3). From these, the dominant epitopes of T and B cells were selected. T cell-dominant epitopes were screened for dominant epitopes using the amount of IFN-γ released from spleen cells and PBMC of tuberculosis, and late-onset hypersensitivity reaction in mice. Through experiments, we found that the polypeptide MPT64 _(152-165)_ is the predominant epitope of T cells(Fig. 9). The absorbance was measured by adding the prepared MPT64 rabbit polyclonal antibody and the PVDF membrane, by coincubation with MPT64 rabbit polyclonal antibody revealed that the dominant epitope of B cells was MPT64_(109-119)_ (Fig. 9).

**TABLE 3:** Sequences of synthetic peptides by MPT64

Peptide	MPT64 peptide sequence	Score	Acid-base property
P0:MPT64	Totally sequence	–^*a*^	–^*a*^
P1:MPT64(4-19)	KIFMLVTAVVLLCCSG	1.308	Alkaline
P2:MPT64(20-32)	VATAAPKTYCEEL	1.308	Acidic
P3:MPT64(38-44)	GQACQIQ	1.078	Neutral
P4:MPT64(51-63)	NINISLPSYYPDQ	1.116	Acidic
P5:MPT64(90-96)	PYELNIT	1.045	Acidic
P6:MPT64(98-106)	ATYQSAIPP	1.076	Neutral
P7:MPT64(109-119)	TQAVVLKVYQN	1.222	Alkaline
P8:MPT64(152-165)	TDPLPVVFPIVQGE	1.217	Acidic
P9:MPT64(171-179)	GQQVSIAPN	1.101	Neutral
P10:MPT64(194-201)	NDGVIFFF	1.093	Acidic
P11:MPT64(182-191)	LDPVNYQNFA	1.074	Acidic
P12:MPT64(204-210)	GELLPEA	1.060	Acidic
P13:MPT64(213-222)	PTQVLVPRSA	1.153	Alkaline

^*a*^ “–” indicates not applicable.

## DISCUSSION

The newly developed diagnostic technology not only requires a large amount of research and development funds (12, 13), but also the research and development of new TB diagnostic means or technology is only to meet the requirements of scientific research, and whether it can play its due detection ability in actual TB diagnosis (14, 15) is worth thinking about. Therefore, improvement or optimization on the basis of the original diagnostic technology or means can not only save a lot of research and development time and capital cost, but also can be well applied in the actual clinical or screening site (16), which is in line with the principle of cost-effectiveness. Therefore, the discovery of new diagnostic targets and how to use them reasonably is of great significance for the diagnosis of tuberculosis today.

The T-SPOT.TB assay relies on ESAT-6 and CFP-10 antigens from the RD1 region for stimulation and is available through various commercial kits (17). However, inconsistent results have been reported, often due to spot count threshold issues (18). For instance, a study in Turkey found that T-SPOT.TB had a specificity of 80.9% and a positive predictive value of 81.3%, which are lower than the TST values of 95.7% and 90.0%, respectively (19). These discrepancies may stem from immature T and B cells, other interfering conditions, or the limited antigen diversity of ESAT-6 and CFP-10, both originating from the same RD region. Research suggests that incorporating multiple antigens from different RD regions can improve the sensitivity and specificity of TB diagnosis (20, 21). Therefore, introducing additional RD-derived protein antigens could enhance IFN-γ secretion in TB patients' PBMCs and mitigate the limitations associated with the T-SPOT assay’s spot threshold.

In this study, we expressed seven highly purified and stable secreted proteins, including the established diagnostic peptides EAST-6 and CFP-10. Additionally, we produced CLE, a fusion of EAST-6 and CFP-10, in *E. coli* for convenience. CLE serves both as a benchmark for new RD region diagnostic targets and as a component for combined tuberculosis diagnosis. ELISA results demonstrated that the purified proteins significantly stimulated IFN-γ release from splenocytes of iH37Rv-immunized mice compared to PBS between 24 and 72 hours. Notably, MPT64, Rv1773, and CLE induced substantial IFN-γ production at 72 hours, with MPT64 showing the highest response. CLE from RD1 is currently the most widely used antigen for TB stimulation. Furthermore, studies have identified Rv1773 as a virulence factor that enhances *M.tb* invasion into macrophages (22). Additionally, Rv1773 is a specific protein of protoBCG, functioning as a transcriptional repressor lost during the *in vitro* evolution of BCG, which enhances the specificity of tuberculosis diagnosis (23). MPT64, an important virulence factor secreted by *M.tb*, regulates host proteins to promote *M.tb* survival and proliferation within the host (24). It is widely used as a diagnostic marker to distinguish the *M.tb* complex from nontuberculous mycobacteria (25). Furthermore, the combination of MPT64 and Rv1986 demonstrated performance comparable to the EAST-6 and CFP-10 antigens in IFN-γ release assays (26). We propose MPT64 as a pivotal target for TB co-diagnosis, especially when combined with the commercially available EAST-6 and CFP-10, to enhance detection accuracy. Recently, MPT64, EAST-6, and CFP-10 have been utilized for diagnosing TB in urine samples as an auxiliary method (27). However, two TB patients were missed, likely due to immunocompromised states (28), early infection stages before antibody production (29), or technical issues with PBMC extraction (30). Additionally, one healthy control may have had latent TB, resulting in a false positive. The consistency test with the T-SPOT.TB assay showed significant agreement (Kappa = 0.805, *P* < 0.001), supporting the use of the MPT64 and CLE combination as a supplement to clinical T-SPOT.TB. Serologically, MPT64-specific IgG4 demonstrated the best diagnostic performance, with 95.6% sensitivity, 91.1% specificity, and an AUC of 0.965, likely due to IgG4’s association with autoimmune inflammatory pulmonary features (31).

It has also been reported that IgG4 is associated with infection and relapse of chronic tuberculosis, demonstrating its role in the serologic diagnosis of tuberculosis (32). Both MPT64 and CLE showed high serologic IgG4 AUC values with strong agreement, indicating that the MPT64-specific antibody subtype IgG4 plays an important auxiliary role in serological diagnosis. Based on this, the optimal working concentrations of MPT64 and CLE were determined using checkerboard ELISA with a gradient dilution method at the nanogram level. The optimal serum dilution was found to be 1:800 for MPT64 and 1:100 for CLE, providing robust support for kit development. Similar to PPD, the MPT64 antigen induced DTH in the skin of iH37Rv preimmunized mice. However, the MPT64 skin test effectively distinguished *M. tuberculosis* infection from BCG inoculation, unlike the TST skin test based on PPD, which cannot differentiate between BCG inoculation and *M.tb* infection. This specificity is likely due to the distinct regions of the MPT64 protein, making MPT64 more suitable for primary screening in high-burden countries. The low specificity of PPD-based TST can lead to false positives, burdening already scarce healthcare resources as suspected TB patients undergo unnecessary imaging studies, resulting in resource wastage. Therefore, MPT64 is more cost-effective for primary tuberculosis screening. Developing a tuberculosis detection kit requires high-purity monoclonal antibodies. High concentrations of MPT64 and CLE polyclonal antibodies were successfully prepared in New Zealand rabbits, achieving titers of 1:102,400, which meet the basic requirements for tuberculosis diagnosis. However, the availability of polyclonal rabbit anti-MPT64 antibodies is limited, and their stable preparation is challenging due to batch-to-batch variations. Replicating antibodies with consistent performance is essential for large-scale use (33). With the successful preparation of high-titer rabbit polyclonal antibodies against MPT64 and CLE, we investigated the B and T cell epitopes of the MPT64 protein. Exploring antigen epitopes aids in the development of TB diagnostic kits, as most commercial kits use antigen peptides as stimuli (34). MPT64 was divided into 13 segments, and MPT64(152-165), fragment 8, was identified as the dominant T cell epitope through IFN-γ release assays and DTH in mouse spleen cells. ELISA and dot blot hybridization revealed MPT64(109-119) as the dominant B cell epitope. Additionally, peptide spatial structure simulation indicated that the dominant epitope peptides had more functional folding and complex spatial structures, which may contribute to their dominance.

In summary, we identified MPT64 as a potential TB diagnostic antigen target by prokaryotic expression of a secreted RD region protein, IFN-γ release assays in PBMCs, and ELISpot assays in TB patients and healthy controls stimulated with MPT64 alone and in combination with the "star antigen" CLE. Compared with the commonly used T-SPOT.TB in clinical practice, the kappa value of the diagnosis was highly consistent. The specific antibody IgG4 against MPT64 and CLE antigens was evaluated in tuberculosis patients and healthy controls, establishing an important adjunct to serological testing. The optimal working concentrations of MPT64 and CLE for ELISA diagnosis were determined and compared with PPD-based TST. The MPT64 protein could stimulate DTH production in iH37Rv preimmunized mice and distinguish BCG inoculation from MTB infection, addressing the limitations of the PPD skin test. Additionally, we explored the dominant T cell and B cell epitopes of the MPT64 protein and its peptides, identifying sequences TDPLPVVFPIVQGE and TDPLPVVFPIVQGE as dominant epitopes, respectively. In the technical optimization of tuberculosis diagnosis, especially in identifying new diagnostic targets, a large number of blood samples from diverse regions, ethnicities, and age groups are required. Therefore, follow-up studies will expand the sample size and enhance the applicability of the MPT64 protein, positioning it as one of the next “star antigens” for tuberculosis diagnosis and supporting TB prevention and control efforts.

Based on the high sensitivity and specificity demonstrated by the MPT64 detection methods in this study and their ability to effectively distinguish tuberculosis infection from BCG vaccination, we propose integrating the MPT64 assay into the current tuberculosis diagnostic workflow. For initial screening, we recommend adopting the MPT64 skin test to exploit its high specificity and thereby reduce false-positive results. In the confirmatory phase, the combined use of MPT64 and CLE ELISpot assays can enhance diagnostic accuracy and enable earlier definitive diagnosis. At the same time, an ELISA detecting MPT64-specific IgG4 antibodies may serve as an auxiliary diagnostic tool and for monitoring treatment response. To drive broad adoption of these approaches, future work should focus on optimizing kit design to lower costs, conducting multicenter studies to validate their effectiveness, and collaborating with relevant agencies to develop formal diagnostic guidelines. Moreover, strengthening educational and promotional efforts is critical to raise awareness of these new methods among both the public and healthcare professionals. Through these measures, MPT64-based assays are poised to play a larger role in tuberculosis diagnosis and treatment monitoring.

### Conclusion

The secretory protein MPT64, expressed in this study, can induce a substantial production of IFN-γ by immune cells and, when used in conjunction with the “star antigen” CLE, offers a novel approach for tuberculosis diagnosis. Tuberculosis patients exhibit significantly elevated levels of specific antibodies, IgG4, against MPT64 and CLE in their serum compared to healthy individuals, making ELISA-based serological diagnostics an important adjunct testing method for tuberculosis. Moreover, MPT64 can distinguish between tuberculosis infection and BCG vaccination in mouse skin tests, demonstrating its potential for effective tuberculosis diagnosis.

### Limitations of the study

There are several limitations to the study. First, the sample size used to determine the sensitivity and specificity of the specific detection method was relatively small, which limited a comprehensive evaluation of the method’s performance and may have introduced random bias into the results. Second, this study primarily focused on patients with active tuberculosis, and investigations into latent tuberculosis infection were insufficient. Future research will expand the sample size and include more diverse populations to further validate the diagnostic efficacy of the MPT64 antigen. Third, the study collected from patients with tuberculosis in this study was all from confirmed patients and was not grouped according to severity. Future studies will further explore whether there are differences in INF-γ levels among patients with different degrees of tuberculosis.

## Data Availability

The data of participants are collected by the authors and uploaded to the database, which makes it easier for the authors to use the data in the process of analyzing data and writing manuscripts. This kind of database system can conveniently shield the data irrelevant to the experiment and effectively protect the privacy of participants. The data that support the findings of this study are available from the First Affiliated Hospital and the Second Affiliated Hospital of Wannan Medical College, China. But restrictions apply to the availability of these data, which were used under license for the current study, and so are not publicly available. Data are, however, available from the authors upon reasonable request and with permission of Wannan Medical College. If someone wants to request the data from this study, they can contact the corresponding author, Yufeng Wen.

## SUPPLEMENTAL MATERIAL

Table S1 spectrum.03381-24-s0001.docx Demographic characteristics of TB group and HD group.

ASM does not own the copyrights to Supplemental Material that may be linked to, or accessed through, an article. The authors have granted ASM a non-exclusive, world-wide license to publish the Supplemental Material files. Please contact the corresponding author directly for reuse.
