# Supplementary material for: MPT64 antigen-induced immune responses as a novel diagnostic tool for tuberculosis
Source: Microbiol Spectr. 2026 Mar 25;14(5):e03381-24. doi: 10.1128/spectrum.03381-24 (PMC13141838; doi:10.1128/spectrum.03381-24)
Supplement: Table S1 — Demographic characteristics of TB group and HD group. [file spectrum.03381-24-s0001.docx]

**Supplementary Table**

Table1 Demographic Characteristics of TB group and HD group

| Characteristic | TB group (n=40) | HD group (n=50) | *P*-value |
| --- | --- | --- | --- |
| Age | 46.5 ± 13.2 | 43.1 ± 11.5 | 0.18 |
| Gender |  |  | 0.35 |
| Male | 25 (63%) | 28 (56%) |  |
| Female | 15 (37%) | 22 (44%) |  |
| BCG vaccination |  |  | 0.78 |
| Yes | 34 (85%) | 43 (87%) |  |
| No | 6 (15%) | 7 (13%) |  |
| Culture grade |  |  | <0.01 |
| Primary school | 12 (29%) | 6 (12%) |  |
| Secondary school | 23 (57%) | 18 (36%) |  |
| University and above | 5 (14%) | 26 (52%) |  |
